# Glucose, Insulin, and Oxygen Interplay in Placental Hypervascularisation in Diabetes Mellitus

**DOI:** 10.1155/2014/145846

**Published:** 2014-09-02

**Authors:** Silvija Cvitic, Gernot Desoye, Ursula Hiden

**Affiliations:** ^1^Department of Obstetrics and Gynecology, Medical University of Graz, Auenbruggerplatz 14, 8036 Graz, Austria; ^2^Institute of Cell Biology, Histology and Embryology, Medical University of Graz, Harrachgasse 21, 8010 Graz, Austria

## Abstract

The placental vasculature rapidly expands during the course of pregnancy in order to sustain the growing needs of the fetus. Angiogenesis and vascular growth are stimulated and regulated by a variety of growth factors expressed in the placenta or present in the fetal circulation. Like in tumors, hypoxia is a major regulator of angiogenesis because of its ability to stimulate expression of various proangiogenic factors. Chronic fetal hypoxia is often found in pregnancies complicated by maternal diabetes as a result of fetal hyperglycaemia and hyperinsulinemia. Both are associated with altered levels of hormones, growth factors, and proinflammatory cytokines, which may act in a proangiogenic manner and, hence, affect placental angiogenesis and vascular development. Indeed, the placenta in diabetes is characterized by hypervascularisation, demonstrating high placental plasticity in response to diabetic metabolic derangements. This review describes the major regulators of placental angiogenesis and how the diabetic environment *in utero* alters their expression. In the light of hypervascularized diabetic placenta, the focus was placed on proangiogenic factors.

## 1. Diabetes in Pregnancy

With the rise of maternal obesity in the Western world, diabetes in pregnancy has become more prevalent and affects a wide range of 3 to 20% of pregnancies. Diabetes in pregnancy comprises several metabolic diseases including gestational diabetes mellitus (GDM), a maternal glucose intolerance that clinically manifests in the 2nd gestational trimester, and type 1 and type 2 diabetes mellitus (T1D, T2D). These diseases cause increased short term fetal risks, foremost increased fetal fat accretion, but they also predispose the offspring to develop metabolic disorders or cardiovascular disease in later life [1, 2]. The placenta as the essential fetal organ nourishing fetal demands is characterized by various changes in morphology and function which may contribute to the fetal complications of diabetes. Particularly the fetoplacental vasculature and endothelium were shown to be susceptible to the diabetic intrauterine environment. Thus, diabetes associated derangements of factors regulating angiogenesis are a major cause of altered placental angiogenesis and hypervascularisation in diabetes [3–9].

## 2. Placental Angiogenesis in Normal Pregnancy

The central role of the placenta in pregnancy is highlighted by the fact that it is the first fetal organ to develop [10]. The placenta is highly vascularized to allow adequate oxygen and nutrient transfer from mother to fetus and back transfer of fetal waste products to the mother. Placental vascular development is tightly regulated by pro- and antiangiogenic factors and is divided into two stages. The first stage of vessel development, vasculogenesis, begins at day 21 after conception when a vascular plexus forms by differentiation of pluripotent mesenchymal progenitor cells into endothelial cells. In the second stage, these first vessels connect and further expand by angiogenesis that continues from day 32 after conception until delivery [11].

## 3. Regulation of Placental Angiogenesis

Various growth factors have been implicated in the regulation and stimulation of angiogenesis in the human placenta with distinct actions throughout gestation. Among these the most prominent are vascular endothelial growth factor (VEGF) and fibroblast growth factor 2 (FGF2). Furthermore erythropoietin (EPA), leptin (LEP), adiponectin (ADIPOQ), placental growth factor (PlGF), angiopoietins (ANGPT), and the insulin/insulin-like growth factor (INS/IGF) system have also been demonstrated to promote placental angiogenesis. Their effects are mediated by specific cell surface receptors on the endothelium. Using gene expression analysis we compared expression levels of receptors for these hormones, growth factors, and cytokines in isolated human fetoplacental endothelial cells (Table 1). Indeed, receptors for all aforementioned proangiogenic factors were expressed with highest levels detected for VEGF, angiopoietin, and adiponectin receptors.

In the placenta, proangiogenic factors targeting the fetoplacental endothelium are often produced by neighboring cells such as trophoblasts, macrophages (Hofbauer cells), and smooth muscle cells. Moreover, proangiogenic factors of placental and fetal origin are also present in the fetal circulation. A subgroup of these factors is sensitive to reduced oxygen levels, and hypoxia is able to stimulate their expression.

### 3.1. Hypoxia

Hypoxia is the major regulator of angiogenesis. In general, it arises when vascular oxygen supply fails to meet metabolic demands. In early pregnancy, however, low oxygen is a physiological condition and drives developmental processes of placenta and embryo proper. During the establishment of the uteroplacental blood flow at the end of the first trimester, fetoplacental oxygen levels steeply rise [12]. Hypoxia affects the expression of multiple proangiogenic factors by regulating their transcription, mRNA stability, and translation. Thus, the low oxygen environment of the early placenta is paralleled by high levels of hypoxia sensitive proangiogenic factors. Mechanisms upregulating their expression under hypoxia are well studied. Whereas global protein synthesis is attenuated under low oxygen by masking the translation initiation sites for ribosomes, specific mRNAs contain alternative, internal ribosome binding sites (IRES) and are preferentially translated. Examples for mRNAs containing IRES include proangiogenic molecules, such as* VEGF*, and hypoxia inducible factors (HIFs), such as* HIF1A*. HIFs subsequently transactivate proangiogenic genes by binding to hypoxia response elements (HRE) in their promoters, introns, or enhancers [13].

### 3.2. VEGF System

The VEGF system constitutes a family of growth factors and their receptors, which are important regulators of blood vessel formation. With the founding factor and most important member, VEGFA (also known as VEGF), this family includes placental growth factor (PlGF), VEGFB, VEGFC, and VEGFD. VEGFs mediate their biological function by a family of protein tyrosine kinase receptors, VEGFR1 (Flt1), VEGFR2 (KDR/Flk1), and Flt4 [14].

In early pregnancy, VEGF is a key driver of placental vascular development and is held responsible for differentiation, growth, and aggregation of the endothelial precursors and formation of the haemangiogenic cords [15]. While villous trophoblasts initiate formation of first placental vessels by secretion of VEGF in the first trimester, it seems that placental macrophages and other stromal cells, for example, smooth muscle cells, take over angiogenic control at later stages of pregnancy [16–18]. VEGFR2 is expressed on the fetoplacental endothelium [19, 20] and mediates mitogenic cellular responses (reviewed in [21]), whereas VEGFR1 localizes to placental macrophages and trophoblast [19] and is an important regulator of VEGFR2 signalling (reviewed in [21]). As for VEGF, the expression of VEGFR2 is most intense in the early stages of pregnancy and declines steeply as pregnancy advances [20, 22].

In contrast to VEGF, the expression of PlGF and the soluble form of VEGFR1 (sFlt1) increase towards term [23–25]. sFlt1 captures free VEGF and prevents its binding to cell surface receptors, Flt1 and KDR, which further attenuates proangiogenic VEGF signaling in the third trimester, when VEGF levels are decreasing. PlGF is expressed in the syncytiotrophoblast [18] and in the smooth muscle cells around the fetoplacental vessels [26] and it stimulates the formation of highly branched capillary networks [27, 28].* In vitro*, PlGF stimulates proliferation of microvascular endothelial cells from human term placenta [29].

Oxygen tension is the key physiological regulator of both VEGF expression and PlGF expression. Hypoxia regulates VEGF transcription at the transcriptional level through HREs and posttranscriptionally via IRESs [18]. A similar posttranscriptional regulation was also demonstrated for PlGF [30].

### 3.3. FGF System

The fibroblast growth factor (FGF) system encompasses 22 members of the heparin-binding fibroblast growth factor family (FGF1 to 10 and FGF16 to 23) and four FGF receptors (FGFR1 to 4) (reviewed in [31]). Basic FGF (bFGF or FGF2) exerts proangiogenic functions on endothelial cells by binding to tyrosine kinase receptors FGFR1 and FGFR2. These functions include stimulation of proliferation, ECM degradation, migration, and modulation of cell-cell interactions (reviewed by [32]). In early placenta, FGF2 is thought to be involved in the recruitment of haemangiogenic progenitor cells, as its expression, similar to VEGF, reaches a maximum in this period of gestation. At term of gestation FGF2 is predominantly expressed in the syncytiotrophoblast, villous stroma, and fetal vessels [33–35]. FGFR1 parallels FGF2 expression [33], thus making the fetoplacental endothelium a target of FGF2 action. Similar to VEGF, FGF2 promoters harbor also both HREs and IRES, which mediate the regulation by hypoxia at the translational and posttranslational levels. The prevailing mechanism seems to be cell type specific [36].

### 3.4. Angiopoietin System

Whilst the VEGF system plays a key role in vessel sprouting and new vessel initiation, angiopoietins have a role in remodeling and maturation phases [37]. Establishment and maintenance of the outer layers of the vessel walls are thought to be controlled, at least in part, by the balance of angiopoietin 1 (ANGPT1) and angiopoietin 2 (ANGPT2), interacting with the angiopoietin receptors Tie1 and Tie2 [38]. Accordingly, ANGPT1 and ANGPT2 mRNA and protein have been detected in perivascular cells of immature intermediate villi [39], where larger arterioles/arteries and venules/veins develop. Both Tie1 and Tie2 mRNA localize to placental trophoblast and vascular endothelium [40, 41]. ANGPT2 belongs to the hypoxia sensitive proangiogenic factors and is regulated by HRE mediated transcriptional induction and by increasing mRNA stability [42, 43].

### 3.5. EG-VEGF/PROKR System

A novel family of angiogenic mitogens with high tissue specificity composed of EG-VEGF/PROK1 and PROK2 and the respective receptors, PROKR1 and PROKR2, has been characterized (reviewed in [44]). Whereas PROK2 mainly associates with the nervous system, EG-VEGF is associated with the reproductive tract including the placenta [45, 46]. EG-VEGF is mainly localized to syncytiotrophoblast with a mild expression in cytotrophoblast [47]. It has a strong vascular bed specificity and promotes proliferation, migration, tube-like formation, and permeability of placental microvascular endothelial cells without affecting angiogenesis in HUVEC [48]. EG-VEGF receptor PROKR1 is abundant in the cytotrophoblast, in the placental microvascular endothelial cells, and in the Hofbauer cells, whereas PROKR2 is expressed in the syncytiotrophoblast, Hofbauer cells, and extravillous trophoblast [47, 49, 50]. The oxygen tension regulates the expression of both EG-VEGF and the receptor PROKR1 at the transcriptional level [47].

### 3.6. Erythropoietin (EPO)

Erythropoietin (EPO) is a hormone that regulates erythropoiesis in an oxygen-dependent manner. Generally, it is produced by perisinusoidal cells in the liver in the fetal and perinatal period and by peritubular interstitial fibroblasts in the kidney in adults [51]. The nonhematopoietic actions of EPO include regulation of angiogenesis during embryonic development [52], mitogenesis, stimulation of circulating endothelial progenitor cells, and cardioprotective effects in the ischemic heart by inhibiting apoptosis of cardiac myocytes [53, 54].

The placenta is also a source of EPO production, because EPO expression has been shown in various trophoblast subpopulations throughout gestation [55]. In addition, the EPO receptor (EPOR) was identified in villous and extravillous cytotrophoblast and syncytiotrophoblast at all gestational stages and also by cells within the villous core including fetoplacental endothelial cells [56].

EPO synthesis is primarily stimulated by hypoxia at the transcriptional level by HIF binding to HRE elements present in the* EPO* gene (reviewed in [57]). In addition, recent evidence suggests hypoxia regulation of EPO expression also at the translational level that does not involve IRES elements [58].

### 3.7. IL6 and TNFA

Interleukin 6 (IL6) and tumor necrosis factor alpha (TNFA) are multifunctional cytokines with main functions in the inflammatory response. In general, IL6 is produced by a variety of cell types, that is, macrophages, muscle cells, fibroblasts, and epithelial cells [59, 60], while TNFA is mainly produced by activated macrophages [61]. Both cytokines are also expressed by the human placenta.

In the first trimester placenta, IL6 expression is moderate and increases up to 4-fold in the third trimester placenta. In early pregnancy IL6 expression is observed in trophoblasts and fetal vessels [62, 63], with potential implication in angiogenesis and vascular remodeling [64]. IL6 acts by binding to an IL6-specific receptor, IL6R, which is expressed by placental trophoblasts and fetoplacental endothelial cells [65]. Hypoxia is reported to increase transcription, translation, and release of IL6 gene product from endothelial and smooth muscle cells [66].

*TNFA* mRNA and protein were also identified in first trimester villi, with strong prevalence in the syncytiotrophoblast and low to none in cytotrophoblasts and villous stromal cells. In term placentas, strong TNFA expression was observed in syncytiotrophoblast and villous stromal cells [67]. TNF signalling is mediated through two receptors, TNFR1 and TNFR2, which are expressed also in fetoplacental cells (reviewed in [68]). Regulation of TNFA transcription is complex and cell type specific [69]. Although* TNFA* mRNA does not contain HRE, chronic hypoxia is able to stimulate TNFA expression [70].

### 3.8. Insulin and IGFs

The insulin/insulin-like growth factor (INS/IGF) system comprises three ligands, insulin and the insulin-like growth factors 1 and 2 (IGF1 and IGF2), three cell-surface receptors that mediate the biological effects of the INS and IGFs, insulin (IR) and the IGF1 and IGF2 receptors (IGF1R and IGF2R), and a family of IGF-binding proteins (IGFBPs) (reviewed in [71]).

Insulin and IGFs are implicated in the regulation of fetal and placental growth and development. IGF1 and IGF2 are synthesized in placental mesenchymal cells, such as macrophages and endothelial cells, with little change throughout gestation. However, while IGF1 is present in the trophoblast compartment at all gestational stages, IGF2 is not found in the syncytiotrophoblast and its expression in villous and extravillous cytotrophoblasts in the first trimester becomes undetectable at term of gestation [72–77].

In early pregnancy, the IGF1R is expressed on the villous cytotrophoblasts, the syncytiotrophoblast, and extravillous cytotrophoblast and additionally on placental macrophages and on fetoplacental endothelium in the third trimester [75]. The IR is localized predominantly at the syncytiotrophoblast with low occurrence in cytotrophoblasts in the first trimester. At term, however, IR is found mainly in the fetoplacental vessels [78]. Absent or low expression of insulin and IGF receptors on fetoplacental endothelium in the first trimester suggests that insulin and IGFs regulate fetoplacental angiogenesis only in later stages of pregnancy but will not contribute to placental vasculogenesis.

### 3.9. Leptin

The adipokine leptin (LEP) regulates food intake and satiety, but it also exerts growth factor and proangiogenic actions. In the placenta it is expressed in cytotrophoblasts, syncytiotrophoblast, amnion, and the fetoplacental endothelial cells [79, 80]. Most of the leptin produced by the placenta is secreted into the maternal circulation and may contribute to the increased maternal leptin levels during pregnancy [81]. Only 5% of leptin is secreted in the fetal circulation [82]. Leptin receptor (LEPR) is markedly expressed during the third trimester of pregnancy and is located primarily on the syncytiotrophoblast [83].* In vitro*, its expression was also noted in fetoplacental endothelial cells [84].

Similar to* VEGF* and* FGF2*, leptin mRNA expression is also regulated by hypoxia, although solely at the transcriptional level by the hypoxia-inducible transcription factor HIF1A [85].

### 3.10. Adiponectin

Adiponectin (ADIPOQ) is an adipokine with insulin sensitizing properties and is proposed to exert angiogenic actions. ADIPOQ gene and protein expression were reported in the human term placenta, with expression primarily in the syncytiotrophoblast [86]. However a recent study contradicts this finding and shows absence of adiponectin in the term placenta [87]. The expression of the adiponectin receptor, ADIPOR2, but not of ADIPOR1, was observed in the cytoplasm of placental cytotrophoblasts and syncytiotrophoblast suggesting that adiponectin may have a physiological function in the placenta during pregnancy [88]. Whether adiponectin receptors are present in the fetoplacental endothelium has not been fully clarified, but high expression levels of ADIPOR1 and ADIPOR2 in isolated fetoplacental cells* in vitro* (Table 1) may suggest their expression also* in vivo*.

## 4. Placental Angiogenesis in Diabetes Mellitus

**Figure 1:**
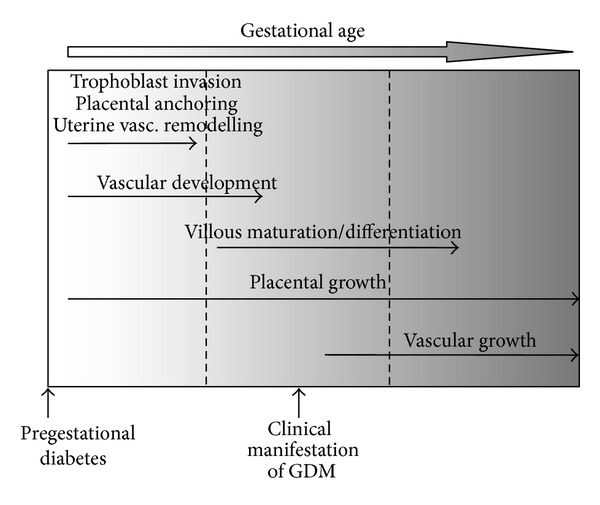
Graphical representation of specific windows of placental development susceptible to metabolic insults of pregestational and gestational diabetes, respectively.

It is well established that maternal diabetes mellitus affects placental vascular development. Depending on the type of diabetes, that is, pregestational (T1D and T2D) or gestational diabetes mellitus (GDM), different windows of placental vascular development are exposed to the diabetic derangements (Figure 1). While pregestational diabetes may affect the entire placental and fetal development, the hyperglycemia of GDM develops during pregnancy and clinically manifests only in the late second trimester. Thus, GDM may have an impact on placental processes occurring in later stages of pregnancy, such as angiogenesis and microvascular remodeling, and will not affect developmental events that occur in early pregnancy, such as vasculogenesis.

This difference in the onset and duration of pregestational diabetes and GDM suggests that the effect of diabetes on placental vascular development and, hence, on the morphology of the placental vascular tree will differ. Indeed, in T1D, three-dimensional power Doppler ultrasound of the fetoplacental vasculature revealed changes in vascular indices already in the first trimester. Thus, T1D affects the placental vasculature already in a period that cannot be affected by GDM. However, the results are conflicting, and both reduced [89] and increased [90] placental vascular indices were reported.

In the third trimester of gestation T1D and GDM lead to similar change of placental vascular structure despite different duration of the diabetic metabolic insult. In T1D the capillary surface area is increased by both longitudinal growth and enhanced branching of villous capillaries [3–7]. Although less investigated, similar observations were made for the placenta in GDM. There are increased capillary branching [8] and capillary surface area [9]. The extent by which capillary branching is increased in T1D and GDM is similar, as reflected by a 2.0-fold versus a 1.8-fold increase in the number of redundant connections per villus, respectively [7, 8]. Further evidence for an impact of maternal diabetes on fetal vascular growth and angiogenesis is provided by findings on longer umbilical cord length in GDM [91] with higher risk for both hyper- and hypocoiled cords [92, 93].

Besides this general finding of placental hypervascularisation, evidence also suggests altered endothelial resistance. Measurements of surface expression of the adherens junctional molecules, vascular-endothelial cadherin (VE-cadherin) and beta-catenin (*β*-catenin), revealed a decreased expression in both T1D [94] and GDM placentas [95]. Moreover, in GDM, the tight junctional molecules, occludin and zonula occludens-1 (ZO1), were also reduced [95]. As these molecules play important roles in angiogenesis and barrier function these results implicate a disturbance of these processes in diabetes. Indeed, microscopic observations of cords after GDM pregnancies revealed rupture of the endothelium paralleled by extravasation of blood within Wharton's jelly [96].

These changes in the fetoplacental vasculature in response to maternal diabetes may imply potential differences also in the vasculature of the fetus proper. In fact, fetuses exposed to diabetes have vessel changes in the iris that resolve after birth [97] and an increased risk in developing cardiovascular defects. The prevalence of cardiovascular defects, however, is different between T1D and GDM. Pregestational diabetes associates with 50% of the investigated cardiac and noncardiac defects in the offspring, while the associations with GDM are weaker and limited to offspring of women with increased prepregnancy BMI [98]. Mechanisms underlying the association of diabetes mellitus with birth defects are not clear. Eriksson and colleagues report a positive correlation between exposure to hyperglycemia during* in utero* development and the risk of congenital malformations in the infants of diabetic mothers [99], suggesting indeed glucose as the driving force of the detrimental effects that diabetes has on the fetus.

## 5. Effect of Maternal Diabetes on Proangiogenic Factors in Placenta and Fetus

The maternal diabetic environment clearly differs between T1D and GDM. However, the emerging metabolic and hormonal changes in the fetus resemble, and it is likely that these changes in the fetus will likewise affect the placental and fetal levels of proangiogenic factors. Some of these changes are summarized in Table 2. The primary origin of fetal derangements in all types of diabetes mellitus is fetal hyperglycaemia resulting from maternal hyperglycaemia. This hyperglycaemia ensues metabolic and hormonal changes in the fetus. Once the fetal pancreas commences to produce and secrete insulin in the late first trimester [100, 101], fetal hyperglycaemia leads to fetal hyperinsulinemia and both stimulate fetal metabolism. Consequently, fetal oxygen demands rise often leading to chronic fetal hypoxia [102–104]. Fetal hypoxia was demonstrated directly by measuring cord blood oxygen [102] and indirectly by measuring cord blood EPO levels and by increased erythropoiesis [103–105].

Because of the strong proangiogenic potency of hypoxia [106] through regulating multiple steps of vascular growth, chronic fetal hypoxia as a consequence of maternal diabetes may thus stimulate placental vasculogenesis and angiogenesis by increasing the growth factors expression in the placenta and fetus. As mentioned before, various proangiogenic factors secreted by the placenta or present in the fetal circulation harbor hypoxia sensitive regulatory sites, that is, IRES or HREs, and may thus be elevated as a result of maternal diabetes. Potential factors regulated by fetal chronic hypoxia in maternal diabetes include VEGF, PlGF, FGF2, EPO, ANG2, and leptin. However, factors of the diabetic environment other than hypoxia may also contribute and modify its effect.

Parallel to its regulation by oxygen, placental VEGF levels are high in the first trimester when oxygen levels are low and decline thereafter towards term of gestation [16–18]. However, in the third trimester, fetal hypoxia as a result of maternal diabetes does not stimulate VEGF expression. In fact, placental expression and fetal cord levels in maternal T1D and GDM are unchanged or even lower than normal [107, 108]. Also PlGF levels are not altered in maternal diabetes [109]. Nevertheless, whole placental tissue from GDM pregnancies contains more VEGFR1 (Flt1) and VEGFR2 (Kdr) [110], which may compensate for the reduced VEGF levels and thus maintain VEGF activity.

Similar to VEGF, also FGF2 levels are higher in the low oxygen environment of the first trimester placenta than in the term placenta [111]. However, in contrast to VEGF, FGF2 is increased in placenta and fetal cord blood in diabetic pregnancies [33, 112]. The presence of an IRES and HRE sequence within its promoter represents a mechanism by which hypoxia increases* FGF2* expression and translation [36].

Hypoxia is also known to induce the expression of EG-VEGF and PROKR1, but their levels were never investigated in diabetes. Nevertheless, since the EG-VEGF and the receptors, PROKR1 and PROKR2, are predominantly expressed during the first trimester of pregnancy the derangement in the level of these factors would affect placental vasculogenesis [47].

Different from the VEGF and FGF2 system, both of which promote sprouting angiogenesis, the angiopoietin system is a major regulator of vessel maintenance and maturation [37]. ANG2 is transcriptionally regulated by hypoxia. This may explain why* ANG2* mRNA expression is increased in the placenta in maternal diabetes [113].

The hormone EPO is also induced by hypoxia and increased in the fetal circulation of diabetic pregnancies at the end of gestation [103]. The EPOR is expressed in fetoplacental vessels [56], making placental endothelial cells a target for EPO action. Experiments in mice revealed that proangiogenic effects of the EPOR are mediated by upregulation of the VEGF/VEGF receptor system [114]. While fetal VEGF levels are unchanged or even lower in maternal diabetes, the VEGFR is increased in the placenta [115]. This would suggest that in a situation of increased fetal oxygen demand fetal and perhaps also placental EPO can promote placental angiogenesis through the VEGF/VEGF receptor system. Although an attractive hypothesis, this awaits demonstration in human.

Collectively, the contribution of hypoxia in altering placental and fetal levels of proangiogenic factors is diverse. While* FGF2* and* EPO* are upregulated in maternal diabetes, the classical hypoxia regulated proangiogenic genes VEGF, PlGF,and ANG2 remain unchanged. This suggests that additional mediators present in the diabetic milieu may also contribute to the diabetes associated hypervascularization. Hyperglycaemia and hyperinsulinemia are such candidates. They may further modify the effect of hypoxia. The contribution of mediators in diabetes in addition to hypoxia is highlighted by differences in placental vascularization between diabetes and other conditions associated with fetal hypoxia, such as high altitude, anemia, or smoking [116]. While increased branching angiogenesis is a common observation, other vascular features, such as total vascular volume, surface area, and capillary length, are increased in maternal diabetes but remain unchanged in the other hypoxic conditions [116].

In addition to hypoxia, hyperglycaemia is one factor that may further impact placental vascular changes, since it is the main reason causing proinflammatory environment and cytokine derangements that will further act on the endothelium. Hyperglycaemia contributes to the generation of reactive oxygen species (ROS) and, thus, to oxidative stress. ROS are generated on one hand by stimulation of the glucose metabolism and the respiratory chain in the mitochondria and on the other hand by the production of advanced glycation end products (AGE). When binding to their receptor (RAGE), AGE induce the formation of ROS as second messengers, a process also termed ROS signaling (reviewed by [117]). As a speculation, this oxidative stress may then contribute to the proinflammatory environment of diabetes and may affect angiogenesis.

Expression of both IL6 and TNFA is sensitive to oxidative stress. Indeed, hyperglycemia stimulates expression of IL6 in trophoblasts [118] and placental expression of IL6 and TNFA is increased [113, 119, 120], but their levels in the fetal circulation are unchanged or even reduced [121, 122]. Thus, TNFA and IL6 may affect placental angiogenesis locally, that is, in a paracrine manner. TNFA and IL6 are not only regulated by the diabetic environment and they are regulators and initiators of the proinflammatory environment of diabetes. Both modulate the expression of adiponectin and leptin. Moreover, TNFA stimulates DNA binding of HIF1 [123] and thus may augment hypoxia induced transcription.

Besides hypoxia and hyperglycaemia, hyperinsulinemia is another hallmark of changes in the fetal circulation associated with maternal diabetes mellitus [5, 124–127]. The potent role of insulin in angiogenesis of fetoplacental endothelial cells has been shown by* in vivo* observations and* in vitro* studies. Fetal cord insulin is positively associated with capillary surface area of the placenta at the end of gestation [5, 128]. In isolated fetoplacental endothelial cells insulin binding to its receptors stimulates several pathways that promote angiogenesis, including the activation of the small GTPase Rac1, eNOS, and expression of the matrix metalloproteinase MT1-MMP [129, 130].

Leptin is regulated by multiple aspects of the fetal diabetic environment. In pregnancies complicated by maternal diabetes mellitus, placental leptin production and fetal plasma leptin is increased [5, 79, 113, 124, 125, 127, 131, 132]. Correlation of fetal insulin with fetal leptin levels suggests that fetal hyperinsulinemia, by stimulating white adipose tissue growth, the main leptin source, leads to higher leptin production in fetal adipose tissue [133]. Additionally, insulin was recently shown to stimulate leptin expression in human placental explants [134]. However, it is unclear whether this leptin will act in a paracrine manner or what proportion is secreted into the fetal circulation. A proinflammatory environment also stimulates leptin secretion. TNFA and IL6 upregulate leptin expression in trophoblasts* in vitro* [135, 136]. Furthermore, correlation of fetal leptin levels with fetal EPO levels in T1D [104] parallels the finding that leptin is regulated by hypoxia through transcriptional induction by HIF1 [85] and can thus represent another manifestation of fetal hypoxia in diabetes mellitus.

Similar to leptin, adiponectin is multifactorially regulated. Both, hyperinsulinemia and hypoxia suppress adiponectin concentrations in adipocytes [137, 138]. Thus, lower levels of adiponectin in the fetal circulation and in the placenta associated with maternal diabetes [85, 86, 139–141] may result from various alterations of the intrauterine environment of diabetes, but reduced levels of the proangiogenic adiponectin suggest that it will not contribute to placental hypervascularisation.

## 6. Conclusion

**Figure 2:**
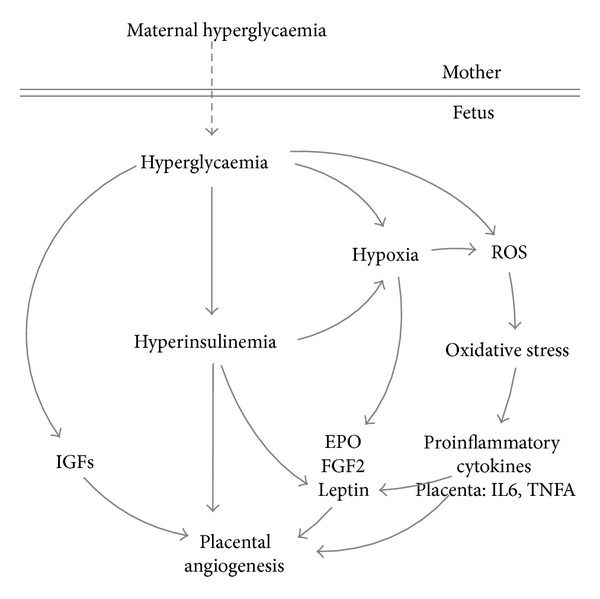
Hypothetic scheme indicating how fetal hyperglycaemia, hyperinsulinemia, and hypoxia may induce metabolic, hormonal, and inflammatory changes that may lead to placental hypervascularisation.

Diabetes mellitus in human pregnancy strongly affects fetoplacental vascular morphology. The fetal diabetic environment resulting from maternal and, hence, fetal hyperglycemia causes fetal hyperinsulinemia and hypoxia. Hypoxia is a key modulator of angiogenesis and works in concert with the diabetic environment to induce placental hypervascularization (Figure 2).

Whether vascular changes occur also in the fetus proper is still unknown. Altered cord blood levels of the majority of proangiogenic factors, as summarized above, however, may suggest potential consequences in the fetus. This speculation is supported by an increased risk for cardiovascular defects [98] and vessel changes in the iris [97] in offspring born to diabetic mothers.

## Figures and Tables

**Table 1:** *In vitro* expression of receptors of proangiogenic factors in the fetoplacental endothelium in normal third trimester human placenta.

Angiogenic factors	Receptors of angiogenic factors			
Gene symbol	Gene symbol	RefSeq ID	Mean intensity	Sd
VEGFA/PlGF	FLT1/VEGFR1	NM_002019	9.5	0.59

VEGFB	KDR/VEGFR2	NM_002253	10.5	1.43

FGF1/FGF2	FGFR1	NM_023110	9.5	0.25
	FGFR2	NM_000141	5.3	0.11
	FGFR3	NM_000142	6.8	0.13
	FGFR4	NM_213647	6.1	0.09

ANGPT1	TIE1	NM_005424	10.8	0.34

ANGPT2	TEK/TIE2	NM_000459	10.1	0.61

EPO	EPOR	NM_000121	7.6	0.15

TNFA	TNFR1	NM_001065	10.8	0.15
	TNFR2	NM_001066	8.6	0.57

IL6	IL6R	NM_000565	6.7	0.28

INS	INSR	NM_000208	6.4	0.70

IGF1	IGF1R	NM_000875	7.5	0.54

IGF2	IGF2R	NM_000876	9.3	0.48

LEP	LEPR	NM_002303	8.51	0.17

ADIPOQ	ADIPOR1	NM_015999	11.6	0.11
	ADIPOR2	NM_024551	9.6	0.18

Mean mRNA signal intensities from arterial (*n* = 9) and venous (*n* = 9) endothelial cells measured by Affymetrix GeneChip Human 1.0 ST arrays (Cvitic et al., unpublished data). Signal intensities range from 1 to 13. Sd: standard deviation.

**Table 2:** Expression and levels of proangiogenic factors in placenta and cord blood in pregnancies complicated by different types of diabetes.

Factor	Type of diabetes	Placenta	Cord blood
VEGF	GDM	↓protein [115]	↓[108]
	T1D	=mRNA [35]	↓[107]

PlGF	GDM	↓protein [115]	↓[108]
	T1D	=mRNA [35]	↓[107]

FGF2	GDM	↑mRNA and protein [112, 132]	↑[112, 142]
	T1D	↑mRNA [33, 111] ↑protein [34] =mRNA [35]	↑[142]

Angiopoietin	GDM	↑mRNA [113]	
	T1D	=mRNA [35]	

Erythropoietin	GDM		↑[103, 108]
	T1D		↑[103, 126]

IL6	GDM	↑mRNA [119]	↓[121]
	T1D		

TNFA	GDM	↑protein [120] ↑mRNA [113] =mRNA [119]	=[122] ↓[121]
	T1D		

Insulin	GDM		↑[124, 125, 127, 143]
	T1D		↑[5, 125, 126]

IGF1	GDM	=mRNA [112] ↓mRNA [113]	↑[112, 144, 145]
	T1D		=[5]
	IDDM		↑[146]
	T2D		=[144]
	D	=mRNA [147]	↑[147]

IGF2	GDM	↑mRNA [113]	↑[148] =[145]
	T1D		
	IDDM		↑[146, 148]
	D	=mRNA [147] ↓peptide [147]	↑[147]

↑ indicates elevated levels, ↓ indicates reduced levels, and = indicates unchanged levels in diabetes.

GDM: gestational diabetes mellitus; T1D: type 1 diabetes; T2D: type 2 diabetes; IDDM: insulin dependent diabetes mellitus without further classification; D: diabetes without further classification.
